# Synchrotron study on the evolution of the radial structural distribution of carbon fiber monofilaments during heat treatment process

**DOI:** 10.1039/d0ra02317e

**Published:** 2020-05-13

**Authors:** Ting You, Wei Liu, Yang Sha, Weiyu Cao

**Affiliations:** State Key Laboratory of Organic-Inorganic Composites, Beijing University of Chemical Technology Beijing China caowy@mail.buct.edu.cn; The Key Laboratory of Education Ministry on Carbon Fiber and Functional Polymer, Beijing University of Chemical Technology Beijing 100029 China; School of Materials, The University of Manchester Oxford Road Manchester M13 9PL UK

## Abstract

Evolution of the radial distribution of a micro-crystalline structure within polyacrylonitrile monofilaments at various temperatures during carbonization was investigated *via* the method of synchrotron wide-angle X-ray diffraction (S-WAXD) and micro-scanning Raman spectroscopy. The result indicated that in the core region of the carbon fiber monofilaments, the *L*_*c*_ is lower while the interplanar spacing (*d*_002_) is higher compared with those in the skin region at 1300 °C. The difference between the skin and core regions in *L*_*c*_ and *d*_002_ constantly decreases as the heat treatment temperature increases. The results of micro-scanning Raman indicated that the micro-crystallites grow faster along the *L*_*a*_ direction, and the graphite degree is higher in the skin region than that in the core region. This is attributed to the fact that the removal of nitrogen element is the domain factor on the growth of a pseudo-graphite crystallite at relatively lower heat treatment temperatures. As the temperature rises, *L*_*c*_ is developed mainly in the core region with the closed packing of a graphite layer, while the crystallites in the skin region grow in both *L*_*a*_ and *L*_*c*_ directions, which are mainly affected by the disordered and boundary graphite structure.

## Abstract 2

Evolution of the radial distribution of the crystalline structure within PAN monofilaments during carbonization was investigated *via* the method of synchrotron diffraction and micro-scanning Raman spectroscopy.

## Graphical abstract

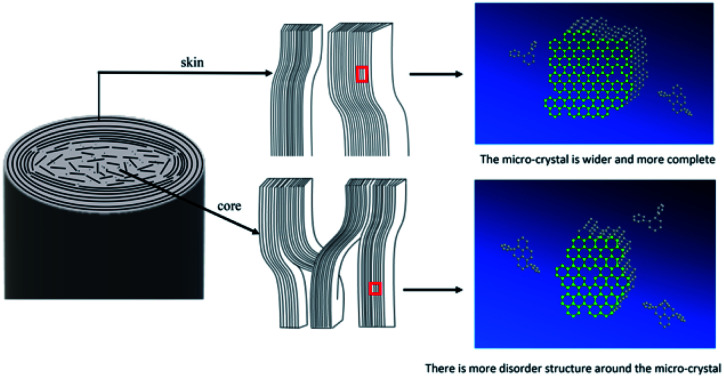

## 1 Introduction

Polyacrylonitrile-based carbon fibers (CFs) have been widely applied as a reinforcement in advanced composites due to their excellent performance.^1,2^ However, compared with the ideal theoretical mechanical properties, carbon fibers have rather relatively low tensile strength and modulus.^3^ For the past few decades, revealing the relationship between microstructures and properties has always been a key subject to serve the purpose of improving the properties of PAN-based carbon fibers. To the best of our knowledge, most of these studies were focused on the monolithic structure of CF. Actually, CF is assumed to be composed of micro-graphite crystallites, in amorphous phase and nano-void. The structural heterogeneity could also be detected between the surface and central parts of a CF monofilament.^4–8^ This kind of structural heterogeneity may result in the distribution of stress along the radial direction within the CF filament, which bears the force load. Therefore, the mechanical properties of carbon fiber were found to be strongly dependent on the radial structural distribution.

The distribution of the crystallite size and inter-planar spacing (*d*_002_) of carbon fiber monofilaments were investigated by Kobayashi *et al.*^6,7^*via* micro-beam synchrotron X-ray measurements. The results indicated that the CF monofilament sample with a modulus of 252 GPa shows almost homogeneous distribution of both the crystallinity and crystallite size along the diameter's direction. However, the CF monofilament sample with a modulus of 445 GPa was found to show an appreciably large difference between the skin and core parts. When the samples were loaded with a tensile force along the fiber axis, the apparent crystallite modulus was found to be different between the samples of different bulk modulus calculated by tracing the (100) and (101) peak positions. This indicated that the stress is heterogeneously distributed in these samples. Therefore, the skin–core structure may be the domain factor that influences the resultant mechanical properties of carbon fibers.

Carbonization is an important process for the manufacturing of carbon fibers. Loidl *et al.*^8^ investigated the effects of carbonization temperature on the local structure and mechanical properties of final carbon fibers. The results of WAXD measurements showed that the preferred orientation of the crystallites rose accompanied with a growth in crystallite size and a decrease in the interlayer spacing. However, for the fiber, the crystallite sizes in the skin are slightly smaller and less oriented, while the opposite is the case observed after heat treatment. Li *et al.*^9^ found that the crystallite size *L*_*a*_ and *L*_*c*_ increased distinctly with the increase in temperature. The micro-void structure of the fibers also changed during the high temperature graphitization stage. Raman spectroscopy, which is sensitive to the sp^2^ and sp^3^ hybridizations of carbon, was used to characterize the microstructure of carbon materials.^10,11^ For CFs, the intensity ratio of two major Raman bands (D and G bands), is proposed as one of the most important parameters to evaluate the microstructural heterogeneity. It was found that the graphitization degree in the skin region changed more rapidly compared with the core since the skin region was found to be more affected by the change in temperature.^12,13^ The formation of radial structural distribution was attributed to the different removing rates of nitrogen element between the skin and core regions as well as heat treatment temperature. Bukalov^14^ divided the radial structure of the fibers into three regions: the skin, which is structurally similar to that of pseudo-graphite, the intermediate heterogeneous near-surface layer, and the core, which is structurally similar to that of high-temperature glassy carbon.

In summary, as the temperature rises, the preferred orientations and crystallite sizes of the carbon fiber increased accompanied by a growth in disorder and boundary carbon structure. However, according to the distribution of heat transferring, the growth rate of crystallites should be different in the radial direction of CF monofilaments. In the present study, synchrotron wide-angle X-ray diffraction and Raman spectroscopy were applied to trace the evolution of the distribution of crystallite sizes and inter-planar spacing along the radial direction within CF monofilaments. The influence of the heat treatment temperature on the formation of structural heterogeneity has also been discussed. The mechanism of the formation and evolution of the heterogeneous radial structure was proposed to contribute to the theory of the relationship between the mechanical properties and structural heterogeneity of carbon fibers.

## 2 Experiments

### 2.1 Preparation of carbon fiber samples

A polyacrylonitrile (PAN) precursor used for stabilization and carbonization was provided from Ningbo Institute of Materials Technology & Engineering, Chinese Academic of Science. A bundle of tow, which consisted of total number of 6000 filaments, was continuously carbonized for 4.5 min under certain temperatures every 100 °C from 1300 °C to 2300 °C after stabilization process. The diameter of individual monofilament of final carbon fibers was measured to be about 5 μm.

### 2.2 Synchrotron wide-angle X-ray diffraction measurements

**Fig. 1:**
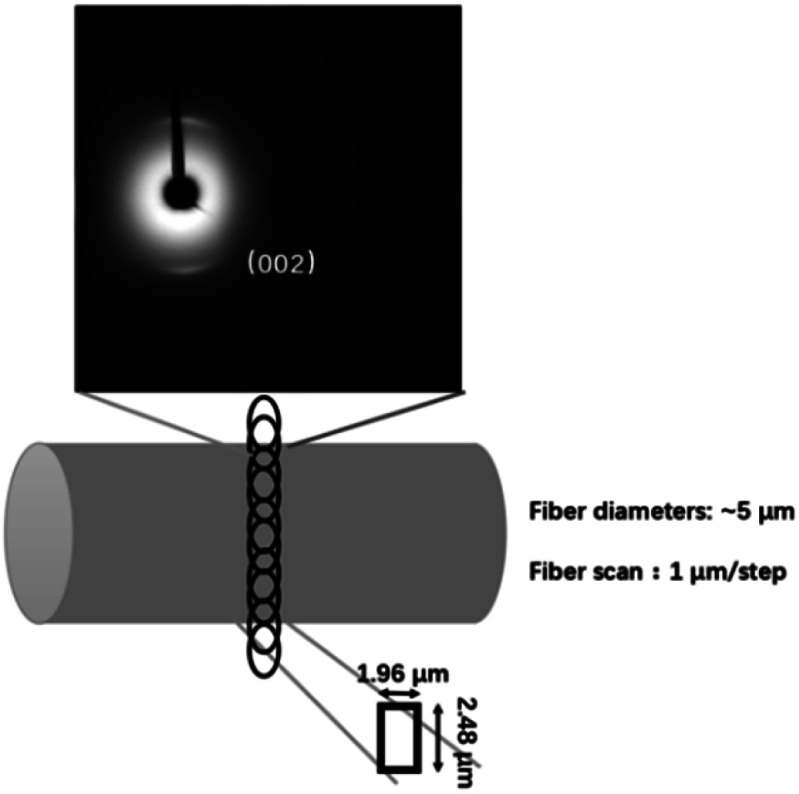
Schematic of the micro-beam synchrotron X-ray diffraction experiments for a single CF monofilament.

Synchrotron wide-angle X-ray diffraction (S-WAXD) experiments were performed at BL15U beamline at Shanghai Synchrotron Radiation Facility (SSRF). The two-dimensional diffraction patterns of the CF monofilament samples were collected using a synchrotron-sourced X-ray beam size of 1.96 μm × 2.48 μm with the wavelength 0.6199 Å. The pixels of detector were 2048 × 2048 and the pixel size was 80 μm. The distance between the sample and the detector was 175.0234 mm, which was calibrated by using the standard, CeO_2_. A monofilament was set horizontally on a sample holder and was irradiated by the synchrotron beam at various radial positions on the fiber with a 1 μm interval, as illustrated in Fig. 1. The exposure time was about 500 s per shot. One-dimensional diffraction profiles were obtained using the Fit2d software *via* cake integration. The average crystallite size, *L*_*c*_ and the inter-planar spacing (*d*_002_) were calculated by the Scherrer's equation (eqn (1)) and Bragg's equation (eqn (2)) respectively, where the peak position (2*θ*) and half-width (*β*) could be obtained from the 1-D profiles.1
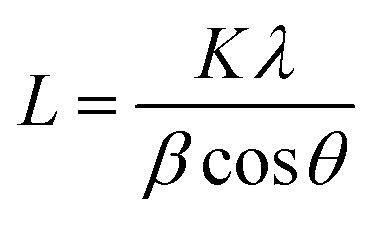

2 2*d* sin *θ* = *nλ*

### 2.3 Raman spectra measurements

The Raman spectra were measured along the direction of the diameter of a CF monofilament using an Invia Reflex Raman Micro-spectrometer, which was equipped with 532 nm wavelength Ar^+^ ion laser. A 100× objective was used to perform a line scanning measurement on the fiber's cross-section. The focused laser beam size was 0.6 μm in diameter. The laser exposure time was 20 s in one shot and the 10 shots were averaged to get one spectrum. The spectra collected were ranged from 800–1800 cm^−1^ Raman shift. The radial distribution of the Raman spectra was obtained by scanning along diameter direction. The ratio of the intensity of the G and D bands, which was used to evaluate the degree of graphitization, was calculated by performing the mixed Lorentz and Gaussian curve fitting of each spectrum. The relationship developed by Tuinstra Keonig:^15,16^3
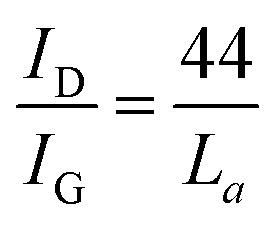


## 3 Results and discussion

### 3.1 Structural heterogeneity of CF monofilaments

**Fig. 2:**
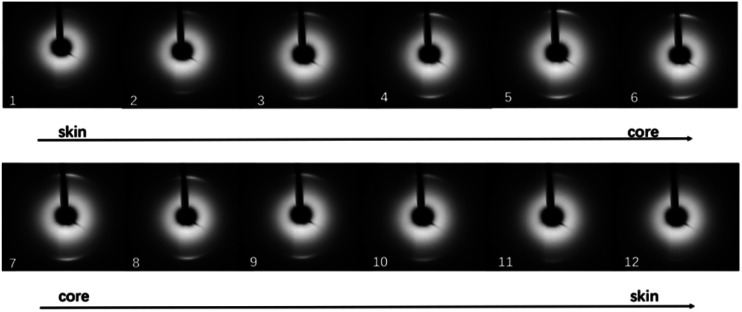
WAXD patterns along the radial direction measured for CF monofilaments heat treated at 2300 °C.

**Fig. 3:**
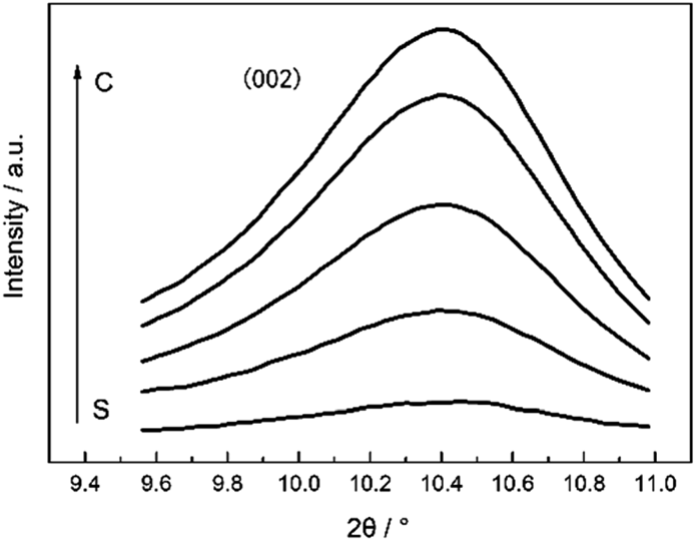
One dimensional WAXD profiles at different radial positions of CF monofilaments heat treated at 2300 °C.

Typical two-dimensional micro-beam synchrotron diffraction patterns at various positions of the carbon fiber monofilament, which was carbonized at 2300 °C, are shown in Fig. 2. Diffraction arcs that come from the (002) Miller plane could be observed in the meridian direction since the sample was set horizontally. It can also be noticed that the position of the (002) diffraction arc shifted towards the beam center with the position closer to the core region of the CF monofilament, which could be distinguished more clearly from the 1-D profile, as shown in Fig. 3. It can be found that the intensity of the (002) diffraction arc was stronger and the Bragg angle shifted to a lower diffraction angle when the position is close to the core.

**Fig. 4:**
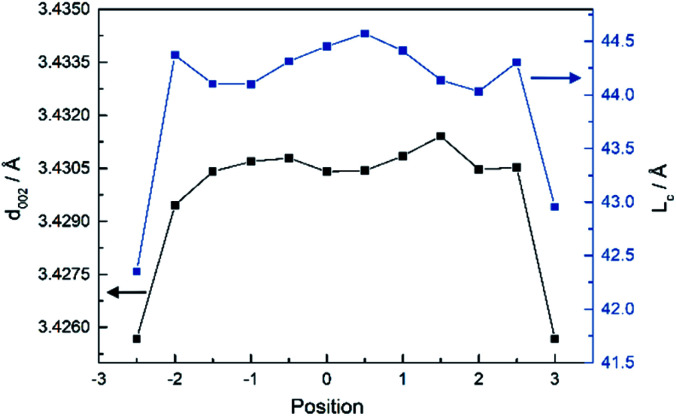
Distribution of *d*_002_ and *L*_*c*_ along the radial direction of CF monofilaments at 2300 °C.

According to calculated results, the *d*_002_ and *L*_*c*_ are higher when the radial position was close to the core at 2300 °C, as shown in Fig. 4. *d*_002_ is the inter-planar stacking spacing. The *L*_*c*_ and *L*_*a*_ are the sizes of the pseudo-graphite crystallite, and are defined as the average crystallite thickness along the normal direction of the (002) Miller plane and the average width of the crystallite along the normal direction of the (100) plane, respectively. The results indicated that the heterogeneity of the crystalline structure of CF monofilaments could be discussed quantitatively and more results from the other samples are shown in the following section.

### 3.2 Evolution of the radial heterogeneity of the CF monofilament with the heat treatment temperature

**Fig. 5:**
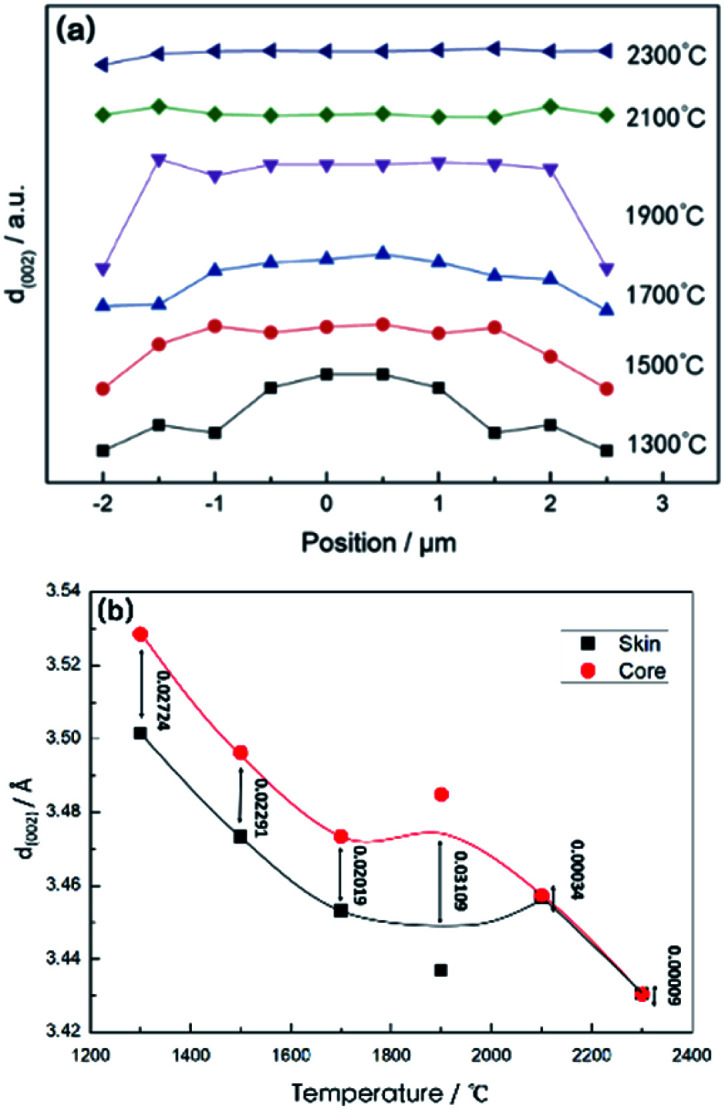
(a) Distribution of *d*_002_ along the radial direction of CF monofilaments at various heat treatment temperatures; (b) the difference of *d*_002_ between skin and core in monofilaments with the increase in the heat treatment temperatures. Note: the position “0” means the center of the filament.

Fig. 5(a) and (b) show the result of a variety of the distribution of *d*_002_ along the radial direction of the monofilaments, heat treated at different temperatures. It can be observed that there is an appreciably large difference in *d*_002_ between the skin and core parts at 1300 °C, which indicated that the inter-planar stacking is denser in the skin than that in the core. The difference in *d*_002_ between the skin and the core reduced as the temperature rose up to 1700 °C, while the difference increased slightly at 1900 °C. Then, the structural distribution tended to be relatively homogeneous with the rise in temperature and *d*_002_ is close to 3.43 Å at 2300 °C. Here, it should be pointed out that the data of the sample treated at 2300 °C is the same as that in Fig. 4. It means that compared to the samples treated at relatively lower temperatures, the radial heterogeneity of the samples treated at relatively higher temperatures is not so obvious although it does exist.

During the carbonization process, non-carbon elements such as nitrogen, hydrogen, oxygen were removed gradually with the increase in the heat treatment temperature. The content of carbon is tended to be more than 99% when the carbonization temperature is higher than 1900 °C. Then, the micro-crystalline structure of the carbon fiber started to rearrange. Therefore, the inter-planar stacking was constantly dense as temperature rose, as shown in Fig. 5(b). However, due to the divergence from the removal speed of non-carbon elements along the radial direction, the gap of the *d*_002_ value between the skin and the core parts of monofilaments decreased and tend to be relatively homogeneous when the temperature reached to 2300 °C, although the radial structural distribution is still present, as shown in Fig. 4.

**Fig. 6:**
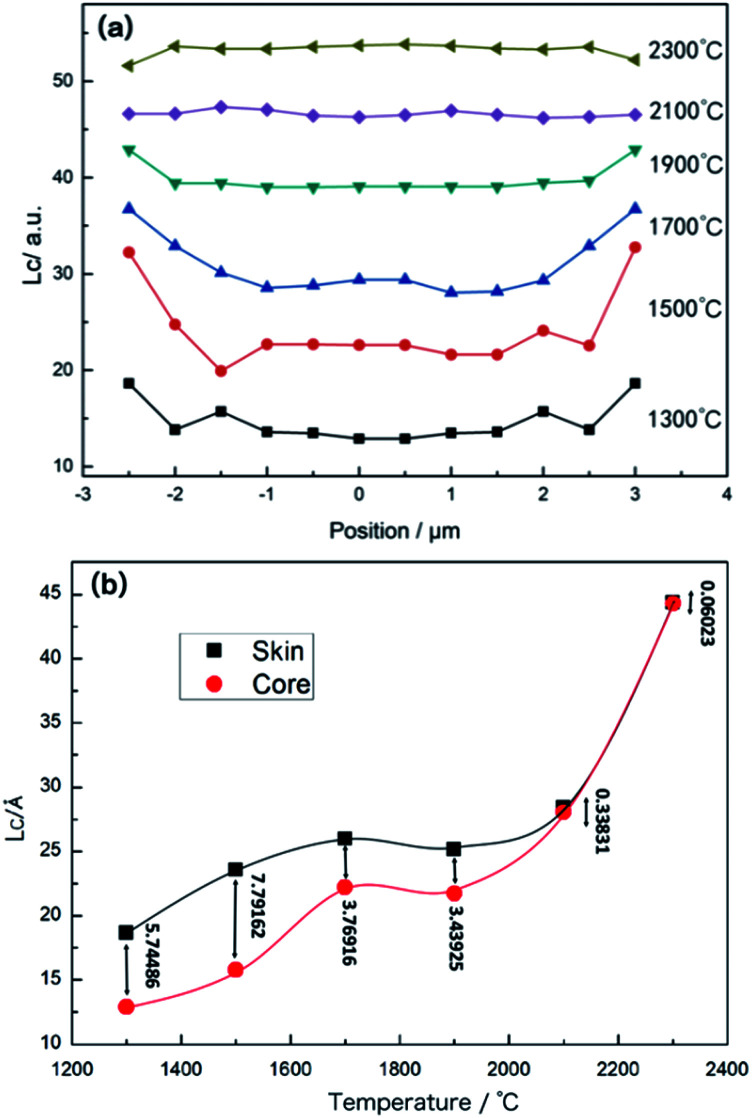
(a) Distribution of *L*_*c*_ of CF monofilaments along the radial direction at various temperatures used for heat treatment; (b) variety of the different *L*_*c*_ between the skin and core regions of CF monofilaments as the heat treatment temperature increases.

Fig. 6(a) shows that the development of *L*_*c*_, which is the thickness of micro-crystals along the (002) direction, with the temperature increase. There is an obvious difference in *L*_*c*_ between the skin and core at 1300 °C, *L*_*c*_ is thicker in the skin as compared to the core region. Then, it increases faster in the core compared to that of skin part and the difference between skin–core decreases with the rise in temperature. As the heat treatment temperature rises above 2100 °C, the distribution of *L*_*c*_ along the radial direction of monofilaments became more homogeneous. The variety of the difference in *L*_*c*_ between the skin and core regions of monofilaments as the treated temperature rose, as shown in Fig. 6(b). The value of *L*_*c*_ increases and the difference along the radial direction gets reduced as the temperature rises, and there is a little difference between the skin and the core at 2400 °C. It could be interpreted as the following: after the removal of the nitrogen element, which almost ended at 1900 °C, the rearrangement of the micro-crystallite was accelerated, results in the tendency of to being relatively homogeneous of *L*_*c*_ along the radial direction. However, this radial distribution could not disappear, as shown in Fig. 4.

**Fig. 7:**
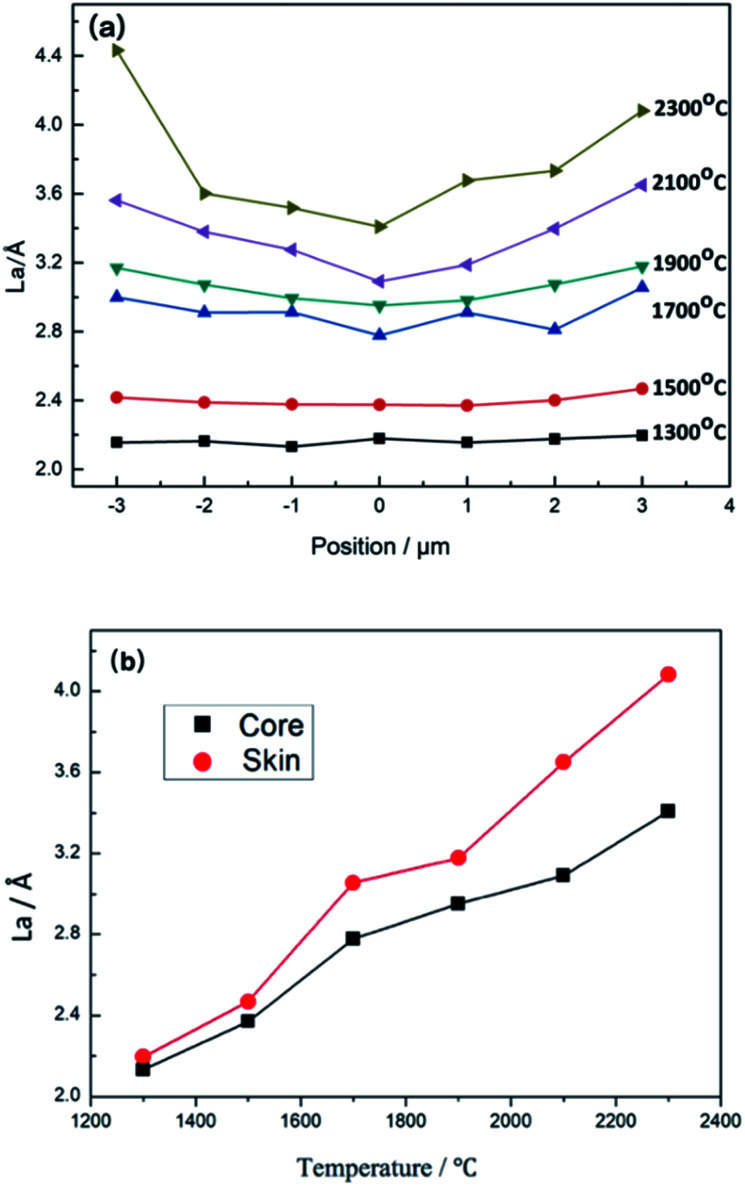
(a) Distribution of *L*_*a*_ along the radial direction of CF monofilaments at various heat treatment temperatures. (b) The difference of *L*_*a*_ between the skin and core regions of CF monofilaments as the heat treatment temperature increased.

**Fig. 8:**
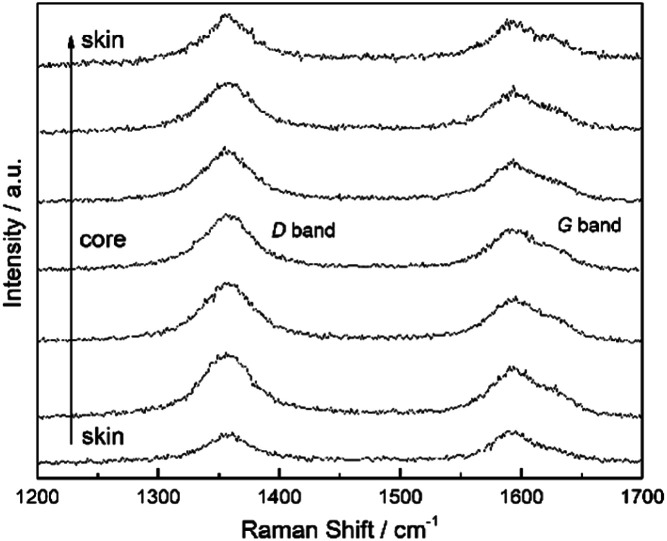
Raman spectra of the CF monofilaments, which were heat treated at 2300 °C.

The distribution of the width of carbon crystallites (*L*_*a*_) along the radial direction of CF monofilaments at various heat treatment temperatures, which was calculated according to eqn (3) based on Raman measurements (typical Raman spectra is shown in Fig. 8), are shown in Fig. 7. It could be observed that *L*_*a*_ is near 2.2 Å and shows a symmetrical homogeneous distribution along the radial direction at 1300 °C. The *L*_*a*_ increases rapidly and still shows a homogeneous distribution as the temperature rises to 1700 °C, and then the *L*_*a*_ increases slightly but the gap between the skin and core increases sharply as the temperature rises further.

The variety of the difference in *L*_*a*_ between the skin and core regions of monofilaments as the treated temperature rose is shown in Fig. 7(b). It can be obviously found that with the increase in the heat treatment temperature, the growth rate of *L*_*a*_ is different in the radial direction, causing a divergent, constant increase between the skin and the core. The difference of *L*_*a*_ between the skin and the core increased slightly below 1900 °C. When the temperature rose above 1900 °C, the gap increased sharply. This result indicated that the growth rate of graphite micro-crystallites is faster than that of the nucleation in the skin with the increase in temperature, resulting in much wider length of the graphite microcrystallites in the skin region than that in the core region.

As mentioned above, the removal of non-carbon elements such as nitrogen element is the domain factor on the growth of the pseudo-graphite crystallite at a relatively lower heat treatment temperature. However, when the carbonization temperature was increased above 1900 °C, the rearrangement of pseudo-graphite crystallites is the key factor that influences the growth of the graphite layers. Based on the skin–core structure inheritance in the carbonization process and chemical reaction, the reduction in *d*_002_ and the increase in *L*_*c*_ mainly occurred in the core region, while the *L*_*a*_ increased mainly in the skin region. Alternatively, this indicated that in the core region of CF monofilaments, the domain is the growth of *L*_*c*_ and closed the stacking of the graphite layer. However, the increase in *L*_*a*_ is due to the domain growth in the skin region.

### 3.3 Distribution of graphitization degree along the radial direction

Fig. 8 is a typical Raman spectrum of the CF monofilaments heat treated at 2300 °C. The characteristic peak at about 1580 cm^−1^ (G band) is attributed to the E_2g_ mode of the stretching vibration of C–C in the graphite lattice. The peak width and the position of the disorder-induced line at 1360 cm^−1^ (D band) are highly sensitive to the defects in the graphite lattice, disarranged and low-symmetrical carbon structure, corresponding to the vibration mode of A_1g_ in the graphite lattice. It could clearly be observed that there is an obvious radial difference in the relative intensity of the D and G bands, which could be a rule of graphite degree.

**Fig. 9:**
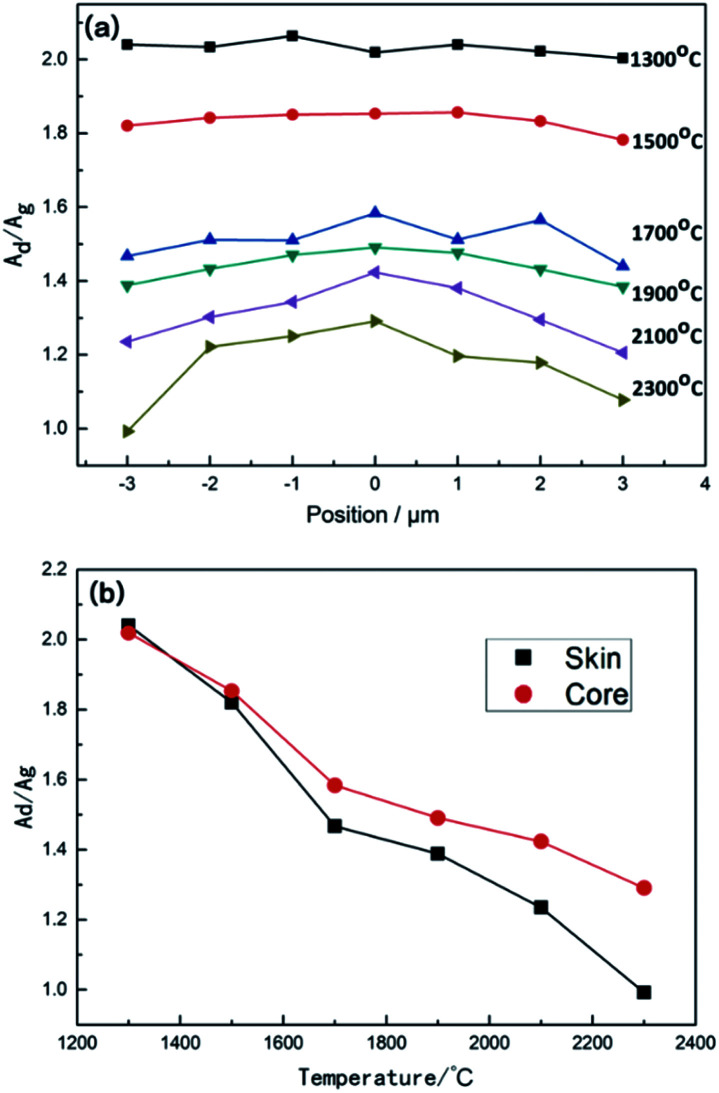
(a) Distribution of the graphitization degree along the radial direction of CF monofilaments at various heat treatment temperatures. (b) The difference of *A*_D_/*A*_G_ between the skin and core regions of CF monofilaments heat-treated at various temperatures.

The calculated results of the distribution of graphitization degree within CF monofilaments at different heat treatment temperatures are shown in Fig. 9(a). The results indicated that the graphitization degree increase with the rise in temperature from 1300 °C to 2300 °C. *A*_D_/*A*_G_ decreases faster when the temperature increased from 1300 °C to 1700 °C, with less heterogeneous distribution of the graphitization degree along the diameter direction of the monofilament. Then, the degree of *A*_D_/*A*_G_ decreases slightly and the difference of *A*_D_/*A*_G_ along the radial direction increases as the carbonization temperature increases until 2300 °C. The difference of *A*_D_/*A*_G_ between the skin and core regions of monofilaments as the treated temperature rises is shown in Fig. 9(b). It shows that *A*_D_/*A*_G_ decreases as the temperature increases, but it decreases more slowly in the core than that of the skin. Then, the divergence between the skin and core increases as the temperature rises.

As mentioned above that during the heat treatment process, the pseudo-graphite crystallites grow along the *L*_*a*_ and *L*_*c*_ directions and the *A*_D_/*A*_G_ decrease from 1300 °C to 1700 °C. When the heat treatment temperature increases furthermore, the growth of the regular crystalline region is slowed down, while the disordered boundary graphite structure increases more quickly. Simultaneously, the growth of the crystallite structure along the *L*_*a*_ direction in the skin region becomes faster than that of the core region. However, it is interesting that the stacking rate of the graphite plane along the *L*_*c*_ direction is almost the same along the radial direction. The inter-relationship between the growth of *L*_*c*_ and the disordered boundary graphite structure in the core parts could be interpreted as following. When the heat treatment temperature is above 1900 °C, the rearrangement rate of pseudo-graphite crystallites became sharper in the core region. Because of this sharp transformation, the graphite micro-crystallite structure along the *L*_*c*_ direction in the core region developed rapidly. Accompanied with the growth of *L*_*c*_, disordered boundary graphite structure also developed. Moreover, because the homogeneous nucleation needs less energy compared to heterogeneous nucleation, the newly generated disordered boundary graphite structure could promote the creation of the graphite micro-crystallite structure. As a result, not only *L*_*c*_ but also D bands in the Raman spectra increase in the core parts.

### 3.4 Evolution model for the radial structural distribution within CF monofilaments with the heat treatment temperature

**Fig. 10:**
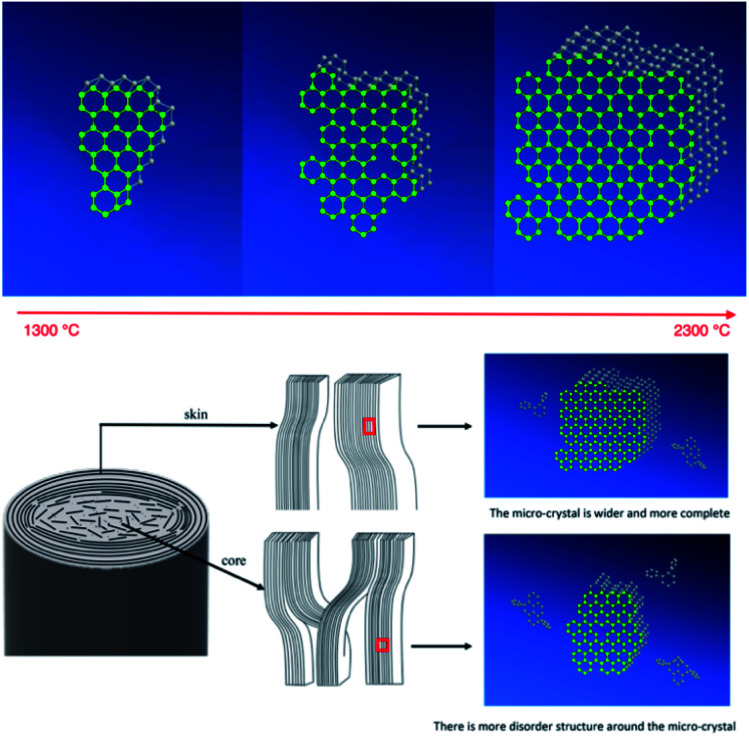
Evolution model for the radial structural distribution with temperature.

The results above indicated that the graphite micro-crystallite was developed along the *L*_*a*_ and *L*_*c*_ directions with the closed stacking of graphite layers as well as constantly decrease in *A*_D_/*A*_G_ with the temperature rise. Furthermore, the growth rate along the radial direction of the graphite micro-crystalline structure is almost the same at relatively lower temperatures. However, in the stage of the rearrangement domain of pseudo-graphite crystallites above 1900 °C, *L*_*c*_ gets developed in the core region accompanying the closed packing of the graphite layer, while the crystallites in the skin region grow in both *L*_*a*_ and *L*_*c*_ directions. Simultaneously, the result that *A*_D_/*A*_G_ is higher in the core could promote the development of *L*_*c*_. Fig. 10 shows the simulated schematic of the development of graphite micro-crystallite structure with the increase in the heat treatment temperature. The crystallites grow along both *L*_*c*_ and *L*_*a*_ directions and *d*_002_ constantly decreases with the increase in temperature. Finally, *d*_002_ and *L*_*c*_ tend to be homogeneous although they still could be detected. However, *L*_*a*_ shows more heterogeneity along the radial direction. It shows that the thickness of micro-crystallites in the core and skin regions are similar, while the width of micro-crystallites in the skin is larger when the heat treatment temperature reaches to 2300 °C. It should be mentioned that after 1900 °C the *A*_D_/*A*_G_ value decreases more slowly in the core compared to the skin region with the increase in temperature. This indicated that there is a more disordered boundary graphite structure in the core region.^16–18^

## 4 Conclusions

The evolution of the micro-crystalline structure of carbon fibers heated from 1300 °C to 2300 °C was investigated *via* synchrotron micro-beam WAXD as well as Raman spectroscopy. The result indicated that the graphite micro-crystallites develop along both *L*_*a*_ and *L*_*c*_ directions with the closed stacking of graphite layers, and the *A*_D_/*A*_G_ value constantly decreases with the increase in the heat treatment temperature. In particular, the radial distribution of the growth rate of crystallites in different directions was clarified. It shows that the growth rate of the graphite micro-crystallite structure along the radial direction is similar at relatively lower temperatures. However, when the heat temperature is above 1900 °C, which is the rearrangement domain of pseudo-graphite crystallites, *L*_*c*_ is developed mainly in the core region with the closed packing of the graphite layer, while the crystallites in the skin region grow in both *L*_*a*_ and *L*_*c*_ directions. This resulted from the higher promotion of the more disordered boundary graphite structure in the core region to the stacking of *L*_*c*_.

## Conflicts of interest

There are no conflicts to declare.

## Supplementary Material
